# The Microbiota-Gut Axis in Premature Infants: Physio-Pathological Implications

**DOI:** 10.3390/cells11030379

**Published:** 2022-01-23

**Authors:** Ilia Bresesti, Silvia Salvatore, Giorgia Valetti, Andreina Baj, Cristina Giaroni, Massimo Agosti

**Affiliations:** 1 Department of Medicine and Surgery, “F. Del Ponte” Hospital, ASST-Settelaghi, University of Insubria, 21100 Varese, Italy; ilia.bresesti@uninsubria.it (I.B.); silvia.salvatore@uninsubria.it (S.S.); 2 Department of Medicine and Surgery, University of Insubria, 21100 Varese, Italy; gvaletti@studenti.uninsubria.it (G.V.); andreina.baj@uninsubria.it (A.B.); cristina.giaroni@uninsubria.it (C.G.)

**Keywords:** preterm infants, microbiota, gut-brain axis

## Abstract

Intriguing evidence is emerging in regard to the influence of gut microbiota composition and function on host health from the very early stages of life. The development of the saprophytic microflora is conditioned by several factors in infants, and peculiarities have been found for babies born prematurely. This population is particularly exposed to a high risk of infection, postnatal antibiotic treatment, feeding difficulties and neurodevelopmental disabilities. To date, there is still a wide gap in understanding all the determinants and the mechanism behind microbiota disruption and its influence in the development of the most common complications of premature infants. A large body of evidence has emerged during the last decades showing the existence of a bidirectional communication axis involving the gut microbiota, the gut and the brain, defined as the microbiota-gut-brain axis. In this context, given that very few data are available to demonstrate the correlation between microbiota dysbiosis and neurodevelopmental disorders in preterm infants, increasing interest has arisen to better understand the impact of the microbiota-gut-brain axis on the clinical outcomes of premature infants and to clarify how this may lead to alternative preventive, diagnostic and therapeutic strategies. In this review, we explored the current evidence regarding microbiota development in premature infants, focusing on the effects of delivery mode, type of feeding, environmental factors and possible influence of the microbiota-gut-brain axis on preterm clinical outcomes during their hospital stay and on their health status later in life.

## 1. Introduction

Preterm births are still associated with various types of adverse outcomes, despite significant improvements in both maternal and postnatal care in the last decades. Although survival rates without severe neuromotor or sensory disabilities are being increasingly reported, children born at less than 34 weeks gestation remain at risk for developmental delay [1,2].

Several factors contribute to infants’ neurodevelopment and health outcomes, and recent research has focused on the role of the bidirectional communication axis between the central nervous system and gastrointestinal tract, the so-called gut-brain axis [3]. In premature infants, interesting data has emerged on the correlation between early gut microbiota colonization and short- and long-term clinical outcomes [4,5,6]. It is now well-established that the intestinal commensal microorganisms are involved in the regulation of many signals between the gut and the brain, creating a more complex microbiota-gut-brain axis [7,8]. Nonetheless, the gut microbiota impacts the preservation of body health homeostasis by regulating several metabolic and cellular functions from the early phases of life, such as the immune system and the defense against pathogenic microorganisms. Such modulation may extend from the gut to the central nervous system (CNS) and is fundamental for brain development and homeostasis [8,9]. Any changes hampering the symbiotic relationship between the microbiota and different cell types composing the enteric microenvironment may have severe consequences, including the development of gut disorders and of behavioral and cognitive diseases [10]. In this narrative review, we aimed to explore the current evidence on the influence of the microbiota-gut-brain axis focusing on preterm infants and their health status later in life.

## 2. Development of the Preterm Infant Gut Microbiota at a Glance

The colonization of an infant’s gut is a crucial step for the development and maturation of the immune system and, consequently, on the well-being of the individual [11]. The traditional view that the fetus is sterile has been questioned by recent evidence demonstrating the presence of microorganisms in amniotic fluid, umbilical cord blood, amniotic membrane and the placenta [12,13,14]. However, this issue still remains greatly debated, since, even in recent studies, the presence of microbiota could not be detected both in placenta samples of either preterm and term deliveries and in fetal meconium [15,16,17]. In contrast, Rackaityte and colleagues were able to detect and grow viable bacterial colonies from fetal meconium and human fetal tissues [18]. Such discrepancies may depend upon the detection method (16s rRNA vs. shotgun sequencing) and different tissues and samples that, overall, can lead to great divergencies in the results. In addition, environmental contamination represents a huge bias that can easily occur, hampering the result reliability.

A general consensus points to the principle that, although intrauterine life is characterized by a restricted, if any, exposure to microbes, an infant’s gastrointestinal tract colonization mostly occurs after birth after exposure to maternal colonic, vaginal and skin microbiota [19,20,21]. However, the establishment of the gut microbiome in preterm neonates still needs to be fully clarified. Although many controversial results have been found [22], some studies have reported differences in gut microbiota composition and development between term and preterm newborns [23,24].

Overall, in preterm infants, the microbial diversity is reduced, with an increased presence of potentially pathogenic bacteria [25,26,27], even if interindividual variations remain elevated. Several studies have attempted to distribute bacterial patterns into five or six more common clusters, also called preterm gut community type, each one characterized by a specific genus of dominance [28,29]. A recurring pattern is featured by initial colonizing facultative anaerobes bacteria, including *Enterobacteria*, *Escherichia coli*, *lactobacilli* and *streptococci*, subsequently replaced by strict anaerobic genera such as *Bifidobacterium*, *Bacteroides*, *Clostridium* and *Eubacterium* by the end of the first week of life [30]. Korpela and colleagues proposed four subsequent phases characterized by the dominance of *Staphylococcus* between 25 and 30 weeks post-menstrual age (PMA), *Enterococcus* from 30 to 35 weeks PMA, *Enterobacter* at 35 weeks PMA and *Bifidobacterium* [31]. The predominant presence of *bifidobacteria* is typical of healthy term infants and is, by far, less represented in preterm neonates and not detected before 30 weeks PMA [31,32]. A prospective study revealed that neonates born <33 weeks gestational age have impaired *Bifidobacterium* colonization and are more predisposed to gut infection and diseases [33].

Evidence from several studies has highlighted the relevance of postnatal age rather than gestational age at birth [26,31,32]. Claud and colleagues also confirmed an age-dependent maturation of the preterm microbiome [27]. They described a shift of preterm microbiome to full-term patterns starting from six weeks of age. In a recent article by Kamal and colleagues, using preterm piglets as a preclinical model, gut microbiota composition and metabolism were affected by preterm birth, especially during the first four weeks of life [34]. On the contrary, after preterm birth, a delayed administration of enteral feeding induced only transitory effects on the gut microbial population and metabolism, suggesting that the early establishment of gut microbiota is influenced by the primary feeding strategy and gestational age at birth, but only the latter yields a clear effect beyond the first week of age.

Among the multiple external determinants that might affect gut colonization, the impact of the mode of delivery is hampered by the low rate of spontaneous vaginal delivery in preterm neonates and, by contrast, by the high rate of antibiotic exposure and possible other confounding factors [35]. A few studies with a small sample size have shown no significant influence of the mode of delivery on the gut microbiota in this population [23,36,37] in contrast with other reports both of preterm and term infants [38,39,40]. Early data showed that, by vaginal delivery, an infant’s gut is colonized with bacteria similar to the mother’s vaginal flora [41,42]. Conversely, in infants born by c-section, the gut microbiota seemed similar to that found on maternal skin and, eventually, on healthcare providers or caregivers and also influenced by the environment [41,42]. Despite this, according to a more recent large cohort study, differences in the gut microbiome could not be significantly attributed to the mode of delivery [43].

Preterm infants often spend the first weeks of life in the Neonatal Intensive Care Unit (NICU), where they often undergo invasive procedures, prolonged antibiotic therapies, delayed enteral feeding and extended parenteral nutrition. All these factors, besides gestational and postnatal age, might influence the gut microbiota development. Noteworthy, the type of feeding (human milk vs. formula) has also been considered, but no firm conclusion has been drawn because of small populations recruited and possible different contributing variables [26,44]. Gregory and colleagues studied three groups of 10 preterm infants <32 weeks gestation fed predominantly with maternal breast milk, donor human milk or infant formula [44]. Their gut microbiome was significantly influenced by birth weight, postnatal age and types of feeding. Infants fed with breast milk presented a greater initial bacterial diversity and a more gradual acquisition of variety than formula-fed infants. The microbiome in the group of infants fed with breast milk were more similar regardless of birth weight in contrast to the microbiome of the formula group, which clustered differently based on birth weight. By adjusting for differences in gut maturity, an ordered succession of microbial phylotypes was observed in breast milk-fed infants, while, in those formula-fed, this succession seemed to be disrupted. In maternal breast milk-fed infants, a consecutive appearance of *Bacillales*, *Lactobacillales*, *Enterobacteriales*, *Clostridiales* and *Bifidobacteriales* was found, whilst formula-fed infants had a longer persistence of Bacillales and Lactobacillales. Moreover, *Clostridiales* were ten times more present in very low birth weight (VLBW) compared to extremely low birth weight (ELBW) infants. Noteworthy, supplementation with pasteurized donor human milk tended to promote a more similar microbiome to breast milk-fed infants and a more rapid increase in bacterial diversity. A study by La Rosa and colleagues involving 58 preterm infants of different degrees of prematurity found that breast milk was associated with an increased proportion of *Gammaproteobacteria* but limited to 28 weeks gestation [32]. In addition, Quigley and colleagues found that preterm infants fed with human donor milk were at a lower risk of developing NEC as compared to preterm formula-fed newborns [45]. This beneficial effect was probably based on the relationship between bacterial populations in the human milk and the microbiota harboring the host gut. This observation suggests that the regulation of milk components may be a strategically important approach to ameliorate preterm newborn health conditions, which may be pursued by changing the maternal diet [46].

The composition of the neonatal gut microbiota is also affected by the timing, duration and type of antibiotic exposure. Intrapartum antibiotic prophylaxis is associated with a decreased diversity and lower lactobacilli and bifidobacteria in the neonatal gut [42]. Lower *Bifidobacterium* has also been shown after postnatal antibiotics use in preterm infants [40,47]. Different antibiotics were found to influence the species variety of the gut microbiota. Gibson and colleagues demonstrated that meropenem, cefotaxime and ticarcillin-clavulanate were significantly associated with reduced gut microbiota diversity, whereas ampicillin, vancomycin and gentamicin were not. They also found an increased risk for opportunistic pathogenic bacteria dominance after antibiotic treatment [48].

Several evidences in the literature have suggested that environmental factors may play a role in preterm gut bacterial establishment [49,50]. Brooks and coworkers performed a metagenomic study of microbes present in 50 preterm newborns and in the environment of the Neonatal Intensive Care Unit (NICU) where they were admitted [51]. The strains present in both sites were *Staphylococcus epidermidis*, *Enterococcus faecalis*, *Pseudomonas aeruginosa* and *Klebsiella pneumoniae*. These species were present in the environment after and often before detection in the preterm gut, showing that a good part of the premature gut population is acquired by microbial exchange between the room and the occupant. In a later study by the same research group, they confirmed that hospitalized preterm infants, in combination with their caregivers, shape the microbiome of NICU rooms [52]. Similar results were presented in a recent multicenter prospective observational study [28] showing that bacterial patterns of VLBW newborns at the fourth postnatal week are influenced by NICU practices. Interestingly, they also pointed out a new promising association between early microbiota composition and 2-year neurological outcomes that may lead to the discovery of a noninvasive, microbiological biomarker.

Last, genetic factors may also be involved in neonatal intestinal microbiota shaping. Interestingly, it has been shown that related twins, although exposed to a NICU environment, maintained a similar gut microbiota [32,53].

## 3. The Microbiota-Gut-Brain Axis in Early Life

The gut-brain axis is a complex network of interconnecting communication pathways between the gut and the brain, encompassing the CNS, the autonomic nervous system (ANS), the enteric nervous system (ENS) and the neuroendocrine and neuroimmune systems [8,54]. In this bidirectional communication axis, the CNS is involved in the coordination and maintenance of the digestive system supporting the control of motility, secretion, nutrient absorption and immune responses [8]. Conversely, signals generated in the gut may affect CNS development, influencing different aspects of behavior in normal and disease states. Different neuronal, immune and hormonal signals take part in this gut-brain interplay, some of which are produced by the commensal microbiota, which is now considered an effective component of the gut-brain axis, defined as the microbiota-gut-brain axis [7,9,55].

The microbiota-gut-brain axis may be of great importance for preterm infants due to their high susceptibility to dysbiosis; however, studies designed to explore the exact pathway/s through which the microbiome specifically affects preterm infant gut and brain development are lacking. To date, the relationship between the gut and brain is mainly associative, based on data from preclinical studies, with the majority of the investigations pointing to neural [56], hormonal and immunological basis mechanisms [3,57,58]. Indeed, the possibility to clarify the neurobiological mechanisms along the microbiota-gut-brain axis underlying the control of preterm infant homeostasis is fundamental, and different molecular pathways may be explored. The correlation between alterations in the gut microbiota and brain function in early postnatal life was demonstrated by resorting to germ-free mice (GF, animals demonstrably free from microbes throughout their lifetime) that displayed altered stress and anxiety responses and memory dysfunction [59,60,61]. If adult, rather than young, GF animals are conventionalized with gut microbiota obtained from specific pathogen-free (SPF) mice, anxiety-like behavior is still evident [59,61], indicating that signals along the microbiota-gut-brain axis may play a role in critical time windows during early postnatal brain development. ENS ontogenesis may also be regulated by gut microbes, since GF mice, at postnatal day 3, displayed an abnormal myenteric plexus compared to SPF mice [62]. The ENS is a complex network constituted of ganglia and interconnecting fiber strands regulating different functions, such as motility, gastric secretion, transport of fluids across the epithelium, blood flow and nutrient absorption, and may undergo significant adaptive changes during postnatal development that depend upon several factors, including not only nutrient composition but also microbial flora composition [63]. It is intriguing to hypothesize that microbial-related changes in the developing gut may also influence brain development via the microbiota-gut-brain axis connections. In this context, attention may be given not only to neuroactive and immunoactive molecules but also to bacterial metabolites, which may represent key signaling pathways along the microbiota-gut-brain axis [64].

Microorganisms harboring the human gut produce a plethora of metabolites, including short-chain fatty acids (SCFAs), tryptophan metabolites and biliary acids, which may influence brain development and function [8,9]. SCFAs, for instance, have been shown to improve gut health by modulating the epithelial barrier integrity, water absorption and mucous production and gut motility [65,66,67]. SCFAs can also directly or indirectly impact on the gut-brain axis by regulating different immune, endocrine, epigenetic and humoral mechanisms [68,69], as well as by directly stimulating vagal and sympathetic nerve signaling [70,71]. In addition to their systemic actions, SCFAs can, to a minimum extent, cross the blood-brain barrier, playing a role as neuroactive substances. In fact, SCFA-free fatty acid receptors 3 and 2 expression in the brain has already been demonstrated [72], giving strength to the potential gut-brain crosstalk. Furthermore, the microbiota has been proposed to impact on early brain development, since GF mice display increased BBB permeability [73,74]. Preclinical studies on germ-free mice indicate that SCFAs may participate in the maintenance of brain homeostasis, influencing learning and cognition [75,76]. Indeed, the supplementation of SCFAs such as butyrate, acetate and propionate in drinking water could ameliorate reward-seeking behaviors and stress responsivity in mice [77]. Tryptophan metabolites, mainly serotonin (5-hydroxytryptamine, 5-HT), kynurenine and microbial-derived indole metabolites, are also recognized as important bioactive molecules influencing gut-brain communication, as described later in this section [78]. Interestingly, it has been demonstrated that GF mice show an increased level of plasmatic 5HT [79], as well as of tryptophan, that was successfully restored to normal after postweaning gut colonization [80]. These observations seem to suggest that the microbiota could both reduce tryptophan availability by expressing tryptophanase or alter host enzymes activity, like IDO or TDO. These alterations in enzymatic activity have already been associated with gastrointestinal disorders [81,82,83], suggesting again the importance of microbiota alterations in disease development.

Noteworthy, the gut microbiota may participate to the production of neuroendocrine and neuroactive molecules [8]. Indeed, the demonstration that bacteria could produce noradrenaline and adrenaline, as proposed about 30 years ago, led to the hypothesis of the existence of a gut-brain bidirectional communication system [84,85]. Since then, several other neuroactive molecules have been recognized as also being of bacterial origin, including a variety of amines such as 5-HT and kynurenine, deriving from tryptophan metabolism, and neurotransmitters, such as glutamate, GABA, dopamine and acetylcholine [86]. There have been reports suggesting that commensal bacteria modulate the 5HT gut levels in vivo by controlling amine biosynthesis, metabolism and transport [78]. In GF mice colons, the biogenic amine levels were significantly lower than in mice colonized with specific pathogen-free or with fecal microbiota [87]. After recolonization, the concentration of free unconjugated, biologically active 5-HT increased, suggesting that bacteria favor free luminal 5-HT accumulation. In GF mice, gut colonization with commensal bacterial normalized plasma levels of tryptophan consequently increase the 5-HT hippocampal levels. Since the biogenic amine cannot enter the BBB, the brain synthesis of 5-HT entirely depends on free circulating tryptophan, suggesting that the microbiota ability to influence CNS serotoninergic transmission relies upon a humoral route [80]. The diversion of tryptophan metabolism from 5-HT towards the kynurenine pathway may favor the manifestation of psychiatric disorders, such as anxiety and major depression [88]. In chronically stressed mice displaying despair behavior, changes of the gut microbiota composition (i.e., reduced Lactobacillus) and increased circulating kynurenine levels were restored after supplementation with *Lactobacillus reuteri*. This latter treatment was associated with an amelioration of behavioral abnormalities, suggesting that microbiota manipulation may help to normalize the metabolism, as well as to favor resilience during stress [89]. Interestingly, in a transgenic mouse model of ASD/(BTBR T^+^ Itpr3^tf^/J mouse) 5-HT, the gut levels were significantly reduced, and this data significantly correlated with the relative abundance of *Bifidobacterium* and *Blautia* [90].

As regards the immunological pathways along the microbiota-gut-brain axis, a few studies have shown that both innate and adaptive immune systems are involved in microbiota-gut-brain axis communication [91,92]. Since dysbiosis, an altered gut microbiota composition and function, is often driven by infection and inflammation, innate and adaptive immunity seems to control the colonization niche of the intestinal microbiota through mechanisms involving the production of antimicrobial peptides or IgA antibodies. In the innate immune system, microbial sensing through pattern recognition receptors (PRRs) is well known, and indeed, Toll-like receptor (TLR)-deficient mice have been shown to harbor a different intestinal microbiota with respect to wild-type animals [93]. Interestingly, the flagellin sensor TLR5 has been suggested to be involved in the prevention of dysbiosis. A similar function is suggested to be carried out by NLR (NOD-like receptors), since NOD1 loss resulted in a general increase of commensal bacteria in mice [94]. In the adaptive immune system, B cells are crucial players in the maintenance of intestinal homeostasis through the production of secretory IgA that can be targeted to specific bacteria, preferentially against those associated with mucosal-proximal colonization and with colitogenic potential [95]. Furthermore, since IgA production is regulated by PD1 signaling, T-helper follicular cells, which highly express PD1 receptors, may participate in microbial regulation [96]. The above mechanisms are involved in the host regulation of the gut microbiota; however, a dysbiotic microbial community, once established, may also substantially affect immune cells both at the local and systemic level, thereby creating a feedback loop in which the host immune system and its microbiota cross-influence each other. This interplay particularly seems to rely on the production of microbial metabolites, such as tryptophan in the case of innate lymphoid cells (ILCs) [97] and short-chain fatty acids in the case of myeloid cells [98] and T-regulatory cells [99]. Of note, microbial metabolite crosstalk with innate immunity starts already during pregnancy, when fetal antibodies mediate the displacement of microbial molecules [100].

Interestingly, preterm pigs at the same postnatal age of term pigs have shown differences in their immune responses, such as much lower counts of erythrocytes, total blood leukocytes and, especially, of neutrophils [101], as well as impaired responses to inflammatory challenges [102]. These findings were also confirmed by clinical data showing a significant deficiency in both innate and adaptive immune responses in preterm infants [103]. These observations overall suggest that preterm neonates may undergo a unique pattern of gut and immune maturation when attempting to adapt, ahead of time, to an abrupt transition from a protected in utero environment to an ex utero environment with independent respiration, enteral feeding and exposure to foreign antigens. In this scenario, it is intriguing to evaluate if changes in the microbiota composition may take part in such immune system adaptations.

Among the neuronal and hormonal pathways conveying signals along the microbiota-gut-brain axis, the vagus nerve and the HPA axis play major roles [8]. In a recent study resorting to transgenic mice Shank3B^−/−^ exhibiting autism spectrum disorder (ASD)-like behaviors, the administration of *Lactobacillus reuteri* ameliorated the animals’ social interactions, supporting adaptive plasticity changes in the ventral tegmental area in a vagus-dependent manner [104]. *Lactobacillus rhamnosus* reduced stress-induced corticosterone and anxiety- and depression-related behaviors through the regulation of GABA receptor expression in the brain, while vagotomy blunted the anxiolytic and antidepressant effects [105]. Besides parasympathetic vagal pathways, preclinical studies carried out on GF rodents after antibiotic and probiotic treatments have shown that the hypothalamic-pituitary-adrenal (HPA) axis and neuroendocrine and immune pathways take part in this microbial-mediated modulation of stress responses [59,106,107].

Given this evidence, an exciting future scenario is represented by the possibility of targeting signaling pathways along the microbiota-gut-brain for potential clinical strategies in preterm infants. In the following sections, we give an overview of the most important studies correlating changes in the microbiota-gut-brain axis in prematurity and the onset and progression of neurological development impairment, necrotizing enterocolitis (NEC) and systemic sepsis.

## 4. Microbiota-Gut-Brain Axis: Implication in Neurodevelopmental Disorders

**Figure 1:**
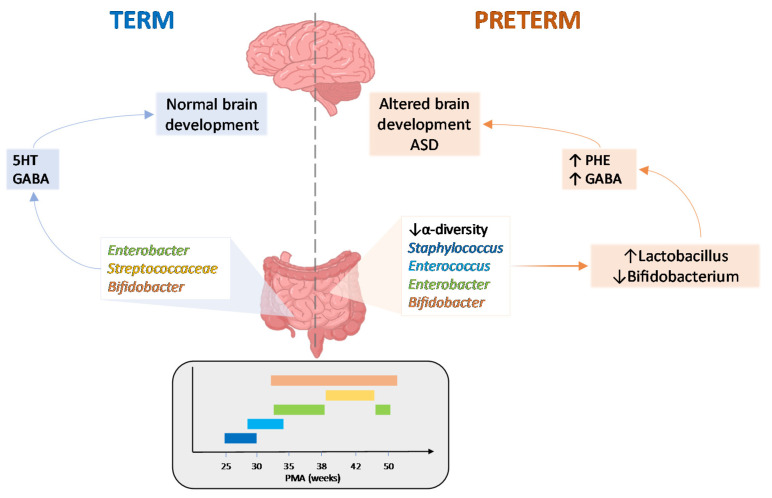
Microbiota-gut-brain axis (MGBA) in healthy and preterm-associated ASD. After birth, the gut microbiota undergoes changes in composition and function in preterm children as compared to children born at term, as depicted in the graph above. The colors in the graph represent the relevant bacterial dominance in the term and preterm groups. In preterm children developing ASD, dysbiosis may influence MGBA communication via the enhanced production of neuroactive molecules such as phenylalanine and GABA impacting early brain development. Abbreviations: 5HT: 5-hydroxitryptamine, serotonin; GABA: gamma aminobutyric acid; PHE: phenylalanine; PMA = post-menstrual age; ASD = autism spectrum disease.

In the last decades, the number of studies showing the ability of gut commensal microorganisms to modulate the gut-brain connecting pathways along the “microbiota-gut-brain axis” during brain development has grown exponentially [108]. This topic is of particular relevance in preterm infants [109], who are at a higher risk of developing neurodevelopmental impairments [110,111], with an increased risk of cerebral palsy and mental disorders. These adverse outcomes may especially involve those babies who are small for gestational age and/or are associated with infection (Figure 1).

Preterm babies are exposed to increased stress levels that, in addition to an immature immune system, may contribute to a higher susceptibility to infections, leading to therapeutic antibiotic treatments. These factors influence neurodevelopment either directly, by favoring inflammatory processes, or indirectly, by changing the composition of the gut microbiome [112]. In this latter context, however, the demonstration of direct effects of a reduced microbiota diversity on neurodevelopment has not yet been sufficiently explored in order to establish a clear-cut correlation. Noteworthy, recent studies have provided evidence that the gut microbiota-immune-brain axis may play a role in brain injury in extremely premature infants [113] and correlate with neurodevelopment at 2 year of age [28].

There is an increasing amount of evidence suggesting that dysbiosis may correlate to the onset of attention deficit hyperactivity disorder (ADHD), autism spectrum disorders (ASD) and other behavioral and psychiatric conditions [114].

Among these neurodevelopmental disorders, ADHD is the most prevalent. Although multiple studies have demonstrated a generally higher incidence of gastrointestinal symptoms in ADHD children compared to the controls, particularly constipation [115], very limited research explicitly connects changes in gut microbiome function and composition to ADHD. Changes in the composition of gut microbiome in early life was demonstrated in children later diagnosed with ADHD [116]. In another study, Prehn-Kristensen [117] found a lower level of alpha diversity in young ADHD patients’ gut commensal bacteria, many of which were involved in GABA production. More interestingly, these authors observed that mothers of ADHD patients showed a reduced gut commensal bacteria alpha diversity as compared to the mothers of healthy controls. In another study, Actinobacteria—in particular, the genus *Bifidobacterium*—were reported to increase at the expense of *Firmicutes* compared to healthy controls [118]. Such an enhancement of *Bifidobacterium* was associated with increased levels of cyclohexadienyl dehydratase, an enzyme involved in the production of phenylalanine, a precursor of dopamine, as well as with reduced neuronal responses to reward anticipation, which constitutes a hallmark of ADHD [118].

More controversial results have been found in regard to ASD. Cao and colleagues did a systematic review concluding that an alteration in the gut microbiome is likely, with no clear evidence with respect to the main phyla alterations [119]. Several, but relatively small, studies have demonstrated altered intestinal microbiota composition related to neurotypical ASD children [10]. However, such data should be interpreted with caution, since individuals with ASD have a higher incidence of antibiotic usage and often receive different diets than healthy children. Adams and colleagues [120] found that children with autism had lower levels of *bifidobacteria* species (−43%, *p* = 0.002) and higher levels of species of *Lactobacillus* (+100%, *p* = 0.00002) and also much lower levels of total neuroactive SCFAs. On the contrary, Wang and colleagues found a 12% increase of SCFAs [121]. Notably, the intracerebroventricular administration of relatively high doses of the SCFA propionic acid to animals resulted in some autistic-like behaviors [122]. From a mechanistic standpoint, SCFAs epigenetically modulate gene expression, promoting histone hyperacetylation by inhibiting histone deacetylases (HDACs), which regulates the brain physiology and disorders such as schizophrenia and stress responses [123].

Interestingly, the incidence of schizophrenia has been positively associated with preterm birth and microbiota changes [124], suggesting the involvement of the early life microbiome in the disease process [125]. The gut microbiota in First Episode Psychosis patients were different from healthy controls. At the family level, *Lactobacillaceae, Halothiobacillaceae, Brucellaceae* and *Micrococcineae* increased, whereas *Veillonellaceae* decreased. At the genus level, *Lactobacillus, Tropheryma, Halothiobacillus, Saccharophagus, Ochrobactrum, Deferribacter* and *Halorubrum* increased, while *Anabaena, Nitrosospira* and *Gallionella* decreased. With the limitation of a small sample size (32 patients), an increased abundance of *Lactobacillus*, in addition to *Bifidobacterium* and *Ascomycota*, was also found in the oropharyngeal microbiome of patients with schizophrenia compared to their healthy controls [126].

### 4.1. The Microbiota-Gut Axis and Necrotizing Enterocolitis

Necrotizing enterocolitis (NEC) is a gastrointestinal complication that mainly affects preterm infants and is a leading cause of morbidity and mortality in this population. NEC is characterized by extensive intestinal tissue necrosis, intestinal villi damage and excessive inflammatory processes in the context of a highly immunoreactive intestine. Since NEC is a gastrointestinal disease, NEC survivors can have long-term health consequences that affect distant organs, such as the brain [6,127,128]. In fact, almost 25% of NEC recovered babies develop microcephaly and serious neurodevelopmental delays [129].

The etiology of NEC still needs to be clarified, although it appears as a multifactorial disease, and enteral feeding, prematurity and altered microbiota represent the major causes for its onset [130,131]. The possibility to clarify the pathophysiological causes of NEC is also hampered by the limitations of the available preclinical models that prevalently rely upon the induction in different animal species of a hypoxic ischemic injury associated with the release of high levels of proinflammatory mediators and/or the chemical excision of Paneth cells and involving molecular pathways dissimilar from those involved in the early onset of NEC in preterm infants.

The majority of infants developing NEC are fed prior to its onset, and feeding is known to strongly influence the gut microbiota composition. The role of bacterial composition and of inappropriate colonization of the gut is strengthened by the evidence that GF animals, as well as preterm piglets administered with broad-spectrum antibiotics, do not develop NEC [132,133,134,135].

A systematic review on intestinal dysbiosis in preterm infants preceding NEC showed an increased relative abundance of *Proteobacteria* and decreased relative abundance of *Firmicutes* and *Bacteroidetes* [136]. There was also evidence that changes in microbiota composition were dependent upon the age of NEC onset. For example, a dominance of *Firmicutes* within the *Clostridia* class, as well as a decrease in *Gammaproteobacteria*, were associated with early-onset NEC occurring within ten days of life [97,98,99]. Late-onset NEC was associated with an increase in *Gammaproteobacteria* and a decrease in *Firmicutes* (especially *Negativicutes*). This result has also been reported after antibiotics administration, thus highlighting the role of antibiotic treatment on the gut colonization of preterm infants [136].

Neither the mode of delivery nor the type of feeding (breast milk or formula) seemed to affect the gut microbiota to such an extent as to correlate with NEC development [136]. The evidence that breastfed infants have a lower risk of NEC may be related to both the maternal milk composition (lactoferrin, polyunsaturated fatty acids, immunoglobulins and immune cells) and to the specific mother–infant microbial content, which is particular to each pair [137].

**Figure 2:**
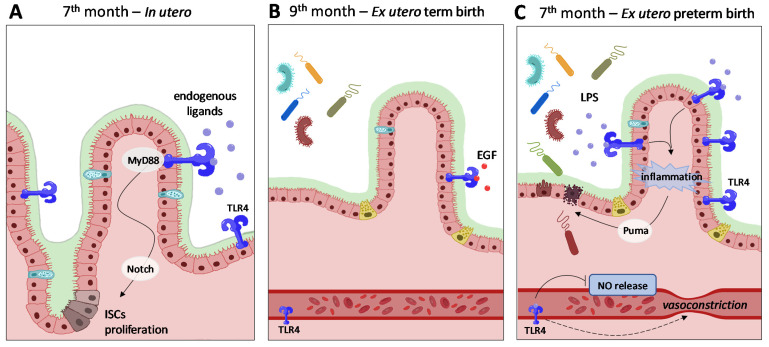
TLR4’s role in pre- and postnatal age and in preterm NEC. (**A**) During the normal gestation activation of TLR4 on epithelial intestinal cells by endogenous ligands, such as HSP class, fibrinogen, fibronectin or hyaluronan, ISC proliferation and differentiation is favored via MyD88 and Notch signaling. (**B**) At term, stimuli associated with vaginal delivery immediately downregulate TLR4 expression in epithelial cells, leading to bacterial tolerance. This protective mechanism is sustained by the inhibitory effect of breast milk−contained EGF on TLR4 signaling. (**C**) In preterm delivery, the tolerance to luminal microorganisms is not acquired, owing to the absence of vaginal delivery stimuli. A high TLR4−mediated inflammatory response to microbial components ensues via MyD88−Puma signaling, leading to the disruption of the epithelial barrier. Bacterial LPS−induced activation of TLR4 on vascular endothelial cells causes the inhibition of NO release and mesenteric vasoconstriction, paving the way for intestinal ischemia and NEC. Abbreviations: ISC = intestinal stem cell; HSP = heat shock protein; EGF = epidermal growth factor; LPS = lipopolysaccharide; NO = nitric oxide.

Preclinical studies of NEC carried out on different animal species, including mice, rats and piglets, have focused on an excessive TLR-mediated response to lipopolysaccharides deriving from the gut bacteria, most often *Gammaproteobacteria*, as the basis of NEC development [138,139]. TLR4 is typically more expressed in a preterm infant’s gut, which is innately more inclined to inflammation, and its levels of expression in the gut immediately fall prior to birth (Figure 2).

In a murine model of NEC, Hackam and colleagues showed that the activation of TLR4 by the dysbiotic bacteria in the premature gut is followed by intestinal epithelium injury and a reduction in the self-repair capability of the intestinal mucosa [140,141]. Furthermore, bacteria translocation across the damaged intestinal barrier into the bloodstream led to the activation of TLR4 on the endothelial lining of premature blood vessels, subsequent vasoconstriction caused by a TLR4-mediated decrease in nitric oxide release and intestinal ischemia, eventually leading to NEC [141]. In line with this theory, the inhibition of TLR4 in the intestinal epithelium showed protection against the development of an experimental NEC in a mouse model subjected to a combination of gavage formula feeds and intermittent hypoxia, while the use of small molecule TLR4 inhibitors prevented NEC in mice, piglet and human tissues ex vivo [141]. The pivotal importance of TLR4 in NEC development has been confirmed by studies on probiotics [138,142], of which it is one of the main sites of action. Specific strains of probiotics are known to promote barrier maturation and function of the intestinal wall, restore altered permeability, regulate immune system, decrease inflammation and impair the growth of pathogenic bacteria [139,143,144]. However, the general administration of probiotic bacteria to prevent NEC in preterm neonates is still debated [145]. According to Siggers and colleagues, probiotics beneficial effects may occur mainly by providing only temporary competitive advantages for certain nonpathogenic resident bacteria that may reduce the potential damaging effects of specific pathogens [130]. According to the last Cochrane review [146], there is an overall beneficial effect from the use of probiotics to prevent NEC. The most commonly used preparations contained *Bifidobacterium* spp., *Lactobacillus* spp., *Saccharomyces* spp. and *Streptococcus* spp. alone or in combinations. The meta-analysis showed that probiotics may reduce the risk of NEC and probably reduce mortality, even though this evidence was assessed as having low or moderate certainty because of the trials’ design limitations. Additionally, probiotics may have little or no effect on severe neurodevelopmental impairment, but the imprecision of accounting for the effect estimate and trial designs prevented a clear conclusion. Sharif and colleagues also concluded that data regarding extremely premature infants are very limited and did not provide evidence on NEC, mortality and infections in this subgroup of preterms.

### 4.2. The Microbiota-Gut-Brain Axis and Sepsis in Premature Infants

Sepsis remains a severe complication of prematurity. Sepsis may occur either in the first seven days of life or later and is defined as “early”-onset sepsis (EOS) or “late”-onset sepsis (LOS), respectively. Despite the recent efforts to implement antibiotic stewardship in NICU, empiric antibiotics are given to pregnant women or preterm infants to reduce the risk of EOS. Thus, paradoxically, a preterm infant’s gut microbiome is disrupted due to the antibiotic therapy, leading to the expansion and dominance of the opportunistic population, then favoring the occurrence of LOS [147]. A few studies have shown a change in the gut microbiome composition before LOS. Mai and colleagues [148] performed a case–control study on preterm ≤32 weeks with LOS. The authors concluded that a distortion in the normal microbiota composition, and not an enrichment of the potential pathogens, is associated with LOS in preterm infants. Carl and coworkers [148] analyzed VLBW neonates. They found that, since birth, invasive E. coli were present in all pre-sepsis stools, but gut colonization with *GBS* and *Serratia marcescens* was detected closer to sepsis. Shaw et al. [149] collected daily fecal samples of preterm neonates <32 weeks gestation, with 22 LOS and 44 matched controls. From the week prior to diagnosis, infants with LOS had higher proportions of fecal aerobes/facultative anaerobes compared to the controls. In 12 cases, the antibiogram of the bloodstream isolate matched that of a component of the fecal microbiota in the samples collected closest to diagnosis. Taft and coworkers [150] studied preterm neonates <29 weeks with LOS and their controls in two NICUs. The gut microbiome disruption occurred before the LOS, but the distortion depended on the postnatal age and the site. The sepsis-causative organism was detected in the stool in 82% of LOS.

According to these findings, it seems evident that several Gram-positive and Gram-negative enteric bacteria have been identified as the major causatives for LOS in preterm infants. Notably, these microorganisms were shown to have intestinal origins rather than translocation from the skin, as demonstrated by stool sampling from LOS cases. Thus, LOS has also been associated with anomalies in the preterm gut microbiome, with reduced bacterial diversity and a higher abundance of *Proteobacteria* and *Firmicutes* but a low *bifidobacteria* presence.

Early life brain development is known to be affected by systemic infection and inflammation, as shown by long-term neurologic impairments among surviving infants [151,152]. At the same time, although this association between premature infection and brain injury has been established, the degree, timing and the underlying mechanism/s are still under investigation. In addition, infection without direct bacterial invasion of the CNS can also lead to brain injury, probably driven by the immaturity of the blood–brain barrier and the inflammation generated by pathogens that activate a local inflammatory response and/or cause direct cytotoxic injury [153]. In addition, infection associated hypoxia/ischemia may lead to proinflammatory microglial activation, which causes the release of inflammatory cytokines such as TNF-α and IL-1β, reactive oxygen and nitrogen species and an increase in the glutamate levels, with subsequent excitotoxicity. The combination of these factors would result in oligodendroglial injury and/or the subsequent inhibition of maturation and myelination, axonal damage and neuronal loss [154]. In a 10-year prospective cohort study on infants born at less than 28 weeks gestational age, investigators found higher adjusted odds for cerebral palsy, autism and epilepsy among infants with histologic chorioamnionitis [155]. In a 5-year follow-up study of 2665 infants born at less than 28 weeks gestation, the investigators found an increased risk of cerebral palsy in survivors of EOS without an increased risk for impaired cognitive outcomes [156]. Mukhopadhyay and colleagues’ results demonstrated that the risk of death/neurodevelopmental impairment for infants with culture-confirmed early-onset sepsis was higher compared with infants without this diagnosis [157].

Even though sepsis pathophysiology in preterm infants is pretty well-understood, its high pathogenic variability and difficulty in pathogenic agent identification still represent important obstacles to the reduction of the mortality rate in preterm infants (around 22%) [158]. From this view, some studies have been directed toward the identification of preventive approaches, such as the DEVANI (Design a Vaccine against Neonatal Infections) project, which was founded by the European Union to identify a maternal immunization program as an effective tool for decreasing the sepsis disease burden [159]. Similarly, a metanalysis study was developed by Kaiser Permanente North California to assess a prediction model for managing neonates at risk of EOS [160]. It incorporates two linked predictive models using a Bayesian approach: the first one considers the probability of EOS development based on gestation, maternal GBS status, duration of membrane rupture, high antepartum maternal temperature and timing and type of intrapartum antibiotics, while the second one evaluates how the baseline risk is modified after neonate medical examination.

## 5. Conclusions

The preterm infant intestinal microbiome is influenced by several factors, beginning from prenatal life. Postnatal changes in the microbiota composition may affect the preterm neonatal course, as well as long-term outcomes. There is still a wide gap in understanding all the contributing factors and the mechanism behind microbiota dysbiosis and its influence in the development of the most common diseases of premature infants and their later consequences. The interplay between the gut and brain warrants further exploration, since it might lead to alternative preventive, diagnostic and therapeutic strategies. In this latter context, future studies are needed to evaluate the efficacy of microbiome−based adjuvant therapies for preterm infants based on the administration of prebiotics, probiotics or even postbiotics, i.e., bacterial−derived metabolites.

## References

[1] Allotey J., Zamora J., Cheong-See F., Kalidindi M., Arroyo-Manzano D., Asztalos E., van der Post J.A.M., Mol B.W., Moore D., Birtles D. (2018). Cognitive, motor, behavioural and academic performances of children born preterm: A meta-analysis and systematic review involving 64,061 children. BJOG Int. J. Obstet. Gynaecol..

[2] Pierrat V., Marchand-Martin L., Arnaud C., Kaminski M., Resche-Rigon M., Lebeaux C., Bodeau-Livinec F., Morgan A.S., Goffinet F., Marret S. (2017). Neurodevelopmental outcome at 2 years for preterm children born at 22 to 34 weeks’ gestation in France in 2011: EPIPAGE-2 cohort study. BMJ.

[3] Lu J., Claud E.C. (2019). Connection between gut microbiome and brain development in preterm infants. Dev. Psychobiol..

[4] Moschopoulos C., Kratimenos P., Koutroulis I., Shah B.V., Mowes A., Bhandari V. (2018). The neurodevelopmental perspective of surgical necrotizing enterocolitis: The role of the gut-brain axis. Mediat. Inflamm..

[5] Lee J.K.F., Tan L.T.H., Ramadas A., Mutalib N.S.A., Lee L.H. (2020). Exploring the Role of Gut Bacteria in Health and Disease in Preterm Neonates. Int. J. Environ. Res. Public Health.

[6] Niemarkt H.J., De Meij T.G., Van Ganzewinkel C.J., De Boer N.K.H., Andriessen P., Hütten M.C., Kramer B.W. (2019). Necrotizing Enterocolitis, Gut Microbiota, and Brain Development: Role of the Brain-Gut Axis. Neonatology.

[7] Baj A., Moro E., Bistoletti M., Orlandi V., Crema F., Giaroni C. (2019). Glutamatergic signaling along the microbiota-gut-brain axis. Int. J. Mol. Sci..

[8] Bistoletti M., Bosi A., Banfi D., Cristina G., Baj A. (2020). The microbiota-gut-brain axis: Focus on the fundamental communication pathways. Progress in Molecular Biology and Translational Science.

[9] Cryan J.F., O’riordan K.J., Cowan C.S.M., Sandhu K.V., Bastiaanssen T.F.S., Boehme M., Codagnone M.G., Cussotto S., Fulling C., Golubeva A.V. (2019). The microbiota-gut-brain axis. Physiol. Rev..

[10] Cryan J.F., Dinan T.G. (2012). Mind-altering microorganisms: The impact of the gut microbiota on brain and behaviour. Nat. Rev. Neurosci..

[11] Staude B., Oehmke F., Lauer T., Behnke J., Göpel W., Schloter M., Schulz H., Krauss-Etschmann S., Ehrhardt H. (2018). The Microbiome and Preterm Birth: A Change in Paradigm with Profound Implications for Pathophysiologic Concepts and Novel Therapeutic Strategies. Biomed. Res. Int..

[12] Aagaard K., Ma J., Antony K.M., Ganu R., Petrosino J., Versalovic J. (2014). The placenta harbors a unique microbiome. Sci. Transl. Med..

[13] Wang X., Buhimschi C.S., Temoin S., Bhandari V., Han Y.W., Buhimschi I.A. (2013). Comparative Microbial Analysis of Paired Amniotic Fluid and Cord Blood from Pregnancies Complicated by Preterm Birth and Early-Onset Neonatal Sepsis. PLoS ONE.

[14] Steel J.H., Malatos S., Kennea N., Edwards A.D., Miles L., Duggan P., Reynolds P.R., Feldman R.G., Sullivan M.H.F. (2005). Bacteria and Inflammatory Cells in Fetal Membranes Do Not Always Cause Preterm Labor. Pediatr. Res..

[15] Walter J., Hornef M.W. (2021). A philosophical perspective on the prenatal in utero microbiome debate. Microbiome.

[16] Kennedy K.M., Gerlach M.J., Adam T., Heimesaat M.M., Rossi L., Surette M.G., Sloboda D.M., Braun T. (2021). Fetal meconium does not have a detectable microbiota before birth. Nat. Microbiol..

[17] de Goffau M.C., Lager S., Sovio U., Gaccioli F., Cook E., Peacock S.J., Parkhill J., Charnock-Jones D.S., Smith G.C.S. (2019). Human placenta has no microbiome but can contain potential pathogens. Nature.

[18] Rackaityte E., Halkias J., Fukui E.M., Mendoza V.F., Hayzelden C., Crawford E.D., Fujimura K.E., Burt T.D., Lynch S.V. (2020). Viable bacterial colonization is highly limited in the human intestine in utero. Nat. Med..

[19] Milani C., Duranti S., Bottacini F., Casey E., Turroni F., Mahony J., Belzer C., Delgado Palacio S., Arboleya Montes S., Mancabelli L. (2017). The First Microbial Colonizers of the Human Gut: Composition, Activities, and Health Implications of the Infant Gut Microbiota. Microbiol. Mol. Biol. Rev..

[20] Lista G., Meneghin F., Bresesti I., Cavigioli F. (2017). Functional nutrients in infants born by vaginal delivery or Cesarean section. La Pediatr. Med. Chir..

[21] Tanaka M., Nakayama J. (2017). Development of the gut microbiota in infancy and its impact on health in later life. Allergol. Int..

[22] Björkström M.V., Hall L., Söderlund S., Håkansson E.G., Håkansson S., Domellöf M. (2009). Intestinal flora in very low-birth weight infants. Acta Pædiatrica.

[23] Grier A., Qiu X., Bandyopadhyay S., Holden-Wiltse J., Kessler H.A., Gill A.L., Hamilton B., Huyck H., Misra S., Mariani T.J. (2017). Impact of prematurity and nutrition on the developing gut microbiome and preterm infant growth. Microbiome.

[24] Groer M.W., Miller E.M., D’Agata A., Ho T.T.B., Dutra S.V., Yoo J.Y., Yee A.L., Gilbert J.A., Dishaw L.J. (2020). Contributors to Dysbiosis in Very-Low-Birth-Weight Infants. J. Obstet. Gynecol. Neonatal Nurs. JOGNN.

[25] Moles L., Gómez M., Heilig H., Bustos G., Fuentes S., de Vos W., Fernández L., Rodríguez J.M., Jiménez E. (2013). Bacterial diversity in meconium of preterm neonates and evolution of their fecal microbiota during the first month of life. PLoS ONE.

[26] Dahl C., Stigum H., Valeur J., Iszatt N., Lenters V., Peddada S., Bjørnholt J.V., Midtvedt T., Mandal S., Eggesbø M. (2018). Preterm infants have distinct microbiomes not explained by mode of delivery, breastfeeding duration or antibiotic exposure. Int. J. Epidemiol..

[27] Claud E.C., Keegan K.P., Brulc J.M., Lu L., Bartels D., Glass E., Chang E.B., Meyer F., Antonopoulos D.A. (2013). Bacterial community structure and functional contributions to emergence of health or necrotizing enterocolitis in preterm infants. Microbiome.

[28] Rozé J.C., Ancel P.Y., Marchand-Martin L., Rousseau C., Montassier E., Monot C., Le Roux K., Butin M., Resche-Rigon M., Aires J. (2020). Assessment of Neonatal Intensive Care Unit Practices and Preterm Newborn Gut Microbiota and 2-Year Neurodevelopmental Outcomes. JAMA Netw. Open.

[29] Stewart C.J., Embleton N.D., Marrs E.C.L., Smith D.P., Fofanova T., Nelson A., Skeath T., Perry J.D., Petrosino J.F., Berrington J.E. (2017). Longitudinal development of the gut microbiome and metabolome in preterm neonates with late onset sepsis and healthy controls. Microbiome.

[30] Jost T., Lacroix C., Braegger C.P., Chassard C. (2012). New Insights in Gut Microbiota Establishment in Healthy Breast Fed Neonates. PLoS ONE.

[31] Korpela K., Blakstad E.W., Moltu S.J., Strømmen K., Nakstad B., Rønnestad A.E., Brække K., Iversen P.O., Drevon C.A., de Vos W. (2018). Intestinal microbiota development and gestational age in preterm neonates. Sci. Rep..

[32] La Rosa P.S., Warner B.B., Zhou Y., Weinstock G.M., Sodergren E., Hall-Moore C.M., Stevens H.J., Bennett W.E., Shaikh N., Linneman L.A. (2014). Patterned progression of bacterial populations in the premature infant gut. Proc. Natl. Acad. Sci. USA.

[33] Butel M.J., Suau A., Campeotto F., Magne F., Aires J., Ferraris L., Kalach N., Leroux B., Dupont C. (2007). Conditions of bifidobacterial colonization in preterm infants: A prospective analysis. J. Pediatr. Gastroenterol. Nutr..

[34] Kamal S.S., Andersen A.D., Krych L., Lauridsen C., Sangild P.T., Thymann T., Nielsen D.S. (2019). Preterm birth has effects on gut colonization in piglets within the first 4 weeks of life. J. Pediatr. Gastroenterol. Nutr..

[35] Hill C.J., Lynch D.B., Murphy K., Ulaszewska M., Jeffery I.B., O’Shea C.A., Watkins C., Dempsey E., Mattivi F., Tuohy K. (2017). Evolution of gut microbiota composition from birth to 24 weeks in the INFANTMET Cohort. Microbiome.

[36] Stewart C.J., Embleton N.D., Clements E., Luna P.N., Smith D.P., Fofanova T.Y., Nelson A., Taylor G., Orr C.H., Petrosino J.F. (2017). Cesarean or vaginal birth does not impact the longitudinal development of the gut microbiome in a cohort of exclusively preterm infants. Front. Microbiol..

[37] Patel A.L., Mutlu E.A., Sun Y., Koenig L., Green S., Jakubowicz A., Mryan J., Engen P., Fogg L., Chen A.L. (2016). Longitudinal survey of microbiota in hospitalized preterm very-low-birth-weight infants. J. Pediatr. Gastroenterol. Nutr..

[38] Wandro S., Osborne S., Enriquez C., Bixby C., Arrieta A., Whiteson K. (2018). The Microbiome and Metabolome of Preterm Infant Stool Are Personalized and Not Driven by Health Outcomes, Including Necrotizing Enterocolitis and Late-Onset Sepsis. Msphere.

[39] Ho T.T.B., Groer M.W., Kane B., Yee A.L., Torres B.A., Gilbert J.A., Maheshwari A. (2018). Dichotomous development of the gut microbiome in preterm infants. Microbiome.

[40] Arboleya S., Sánchez B., Milani C., Duranti S., Solís G., Fernández N., De Los Reyes-Gavilán C.G., Ventura M., Margolles A., Gueimonde M. (2015). Intestinal Microbiota Development in Preterm Neonates and Effect of Perinatal Antibiotics. J. Pediatr..

[41] Penders J., Thijs C., Vink C., Stelma F.F., Snijders B., Kummeling I., Van Den Brandt P.A., Stobberingh E.E. (2006). Factors influencing the composition of the intestinal microbiota in early infancy. Pediatrics.

[42] Dominguez-Bello M.G., Costello E.K., Contreras M., Magris M., Hidalgo G., Fierer N., Knight R. (2010). Delivery mode shapes the acquisition and structure of the initial microbiota across multiple body habitats in newborns. Proc. Natl. Acad. Sci. USA.

[43] Chu D.M., Ma J., Prince A.L., Antony K.M., Seferovic M.D., Aagaard K.M. (2017). Maturation of the infant microbiome community structure and function across multiple body sites and in relation to mode of delivery. Nat. Med..

[44] Gregory K.E., Samuel B.S., Houghteling P., Shan G., Ausubel F.M., Sadreyev R.I., Walker W.A. (2016). Influence of maternal breast milk ingestion on acquisition of the intestinal microbiome in preterm infants. Microbiome.

[45] Quigley M., Embleton N.D., McGuire W. (2019). Formula versus donor breast milk for feeding preterm or low birth weight infants. Cochrane Database Syst. Rev..

[46] Buffet-Bataillon S., Bellanger A., Boudry G., Gangneux J.P., Yverneau M., Beuchée A., Blat S., Le Huërou-Luron I. (2021). New Insights Into Microbiota Modulation-Based Nutritional Interventions for Neurodevelopmental Outcomes in Preterm Infants. Front. Microbiol..

[47] Chernikova D.A., Madan J.C., Housman M.L., Zain-ul-abideen M., Lundgren S.N., Morrison H.G., Sogin M.L., Williams S.M., Moore J.H., Karagas M.R. (2018). The premature infant gut microbiome during the first 6 weeks of life differs based on gestational maturity at birth. Pediatr. Res..

[48] Gibson M.K., Wang B., Ahmadi S., Burnham C.A.D., Tarr P.I., Warner B.B., Dantas G. (2016). Developmental dynamics of the preterm infant gut microbiota and antibiotic resistome. Nat. Microbiol..

[49] Palmer C., Bik E.M., DiGiulio D.B., Relman D.A., Brown P.O. (2007). Development of the Human Infant Intestinal Microbiota. PLoS Biol..

[50] Aguilar-Lopez M., Dinsmoor A.M., Ho T.T.B., Donovan S.M. (2021). A systematic review of the factors influencing microbial colonization of the preterm infant gut. Gut Microbes.

[51] Brooks B., Olm M.R., Firek B.A., Baker R., Thomas B.C., Morowitz M.J., Banfield J.F. (2017). Strain-resolved analysis of hospital rooms and infants reveals overlap between the human and room microbiome. Nat. Commun..

[52] Brooks B., Olm M.R., Firek B.A., Baker R., Geller-McGrath D., Reimer S.R., Soenjoyo K.R., Yip J.S., Dahan D., Thomas B.C. (2018). The developing premature infant gut microbiome is a major factor shaping the microbiome of neonatal intensive care unit rooms. Microbiome.

[53] Stewart C.J., Marrs E.C.L., Nelson A., Lanyon C., Perry J.D., Embleton N.D., Cummings S.P., Berrington J.E. (2013). Development of the Preterm Gut Microbiome in Twins at Risk of Necrotising Enterocolitis and Sepsis. PLoS ONE.

[54] Mayer E.A. (2011). Gut feelings: The emerging biology of gut–brain communication. Nat. Rev. Neurosci..

[55] Bosi A., Banfi D., Bistoletti M., Giaroni C., Baj A. (2020). Tryptophan Metabolites Along the Microbiota-Gut-Brain Axis: An Interkingdom Communication System Influencing the Gut in Health and Disease. Int. J. Tryptophan Res..

[56] Bonaz B., Bazin T., Pellissier S. (2018). The vagus nerve at the interface of the microbiota-gut-brain axis. Front. Neurosci..

[57] Berer K., Mues M., Koutrolos M., AlRasbi Z., Boziki M., Johner C., Wekerle H., Krishnamoorthy G. (2011). Commensal microbiota and myelin autoantigen cooperate to trigger autoimmune demyelination. Nature.

[58] Sampson T.R., Mazmanian S.K. (2015). Control of Brain Development, Function, and Behavior by the Microbiome. Cell Host Microbe.

[59] Sudo N., Chida Y., Aiba Y., Sonoda J., Oyama N., Yu X.-N., Kubo C., Koga Y. (2004). Postnatal microbial colonization programs the hypothalamic-pituitary-adrenal system for stress response in mice. J. Physiol..

[60] Gareau M.G., Wine E., Rodrigues D.M., Cho J.H., Whary M.T., Philpott D.J., MacQueen G., Sherman P.M. (2011). Bacterial infection causes stress-induced memory dysfunction in mice. Gut.

[61] Heijtz R.D., Wang S., Anuar F., Qian Y., Björkholm B., Samuelsson A., Hibberd M.L., Forssberg H., Pettersson S. (2011). Normal gut microbiota modulates brain development and behavior. Proc. Natl. Acad. Sci. USA.

[62] Collins J., Borojevic R., Verdu E.F., Huizinga J.D., Ratcliffe E.M. (2014). Intestinal microbiota influence the early postnatal development of the enteric nervous system. Neurogastroenterol. Motil..

[63] Giaroni C. (2015). Purinergic signalling and development of the autonomic nervous system. Auton. Neurosci. Basic Clin..

[64] Bourassa M.W., Alim I., Bultman S.J., Ratan R.R. (2016). Butyrate, neuroepigenetics and the gut microbiome: Can a high fiber diet improve brain health?. Neurosci. Lett..

[65] Arnoldussen I.A.C., Wiesmann M., Pelgrim C.E., Wielemaker E.M., van Duyvenvoorde W., Amaral-Santos P.L., Verschuren L., Keijser B.J.F., Heerschap A., Kleemann R. (2017). Butyrate restores HFD-induced adaptations in brain function and metabolism in mid-adult obese mice. Int. J. Obes..

[66] Val-Laillet D., Guérin S., Coquery N., Nogret I., Formal M., Romé V., Le Normand L., Meurice P., Randuineau G., Guilloteau P. (2018). Oral sodium butyrate impacts brain metabolism and hippocampal neurogenesis, with limited effects on gut anatomy and function in pigs. FASEB J..

[67] van de Wouw M., Boehme M., Lyte J.M., Wiley N., Strain C., O’Sullivan O., Clarke G., Stanton C., Dinan T.G., Cryan J.F. (2018). Short-chain fatty acids: Microbial metabolites that alleviate stress-induced brain-gut axis alterations. J. Physiol..

[68] Barichello T., Generoso J.S., Simões L.R., Faller C.J., Ceretta R.A., Petronilho F., Lopes-Borges J., Valvassori S.S., Quevedo J. (2015). Sodium Butyrate Prevents Memory Impairment by Re-establishing BDNF and GDNF Expression in Experimental Pneumococcal Meningitis. Mol. Neurobiol..

[69] Stilling R.M., van de Wouw M., Clarke G., Stanton C., Dinan T.G., Cryan J.F. (2016). The neuropharmacology of butyrate: The bread and butter of the microbiota-gut-brain axis?. Neurochem. Int..

[70] Borre Y.E., O’Keeffe G.W., Clarke G., Stanton C., Dinan T.G., Cryan J.F. (2014). Microbiota and neurodevelopmental windows: Implications for brain disorders. Trends Mol. Med..

[71] Bistoletti M., Caputi V., Baranzini N., Marchesi N., Filpa V., Marsilio I., Cerantola S., Terova G., Baj A., Grimaldi A. (2019). Antibiotic treatment-induced dysbiosis differently affects BDNF and TrkB expression in the brain and in the gut of juvenile mice. PLoS ONE.

[72] Brown A.J., Goldsworthy S.M., Barnes A.A., Eilert M.M., Tcheang L., Daniels D., Muir A.I., Wigglesworth M.J., Kinghorn I., Fraser N.J. (2003). The Orphan G protein-coupled receptors GPR41 and GPR43 are activated by propionate and other short chain carboxylic acids. J. Biol. Chem..

[73] Stolp H.B., Dziegielewska K.M., Ek C.J., Potter A.M., Saunders N.R. (2005). Long-term changes in blood–brain barrier permeability and white matter following prolonged systemic inflammation in early development in the rat. Eur. J. Neurosci..

[74] Braniste V., Al-Asmakh M., Kowal C., Anuar F., Abbaspour A., Tóth M., Korecka A., Bakocevic N., Guan N.L., Kundu P. (2014). The gut microbiota influences blood-brain barrier permeability in mice. Sci. Transl. Med..

[75] Frost G., Sleeth M.L., Sahuri-Arisoylu M., Lizarbe B., Cerdan S., Brody L., Anastasovska J., Ghourab S., Hankir M., Zhang S. (2014). The short-chain fatty acid acetate reduces appetite via a central homeostatic mechanism. Nat. Commun..

[76] Nagatsu T. (1995). Tyrosine hydroxylase: Human isoforms, structure and regulation in physiology and pathology. Essays Biochem..

[77] Zhang S., Jin Y., Zeng Z., Liu Z., Fu Z. (2015). Subchronic Exposure of Mice to Cadmium Perturbs Their Hepatic Energy Metabolism and Gut Microbiome. Chem. Res. Toxicol..

[78] Banfi D., Moro E., Bosi A., Bistoletti M., Cerantola S., Crema F., Maggi F., Giron M.C., Giaroni C., Baj A. (2021). Impact of Microbial Metabolites on Microbiota–Gut–Brain Axis in Inflammatory Bowel Disease. Int. J. Mol. Sci..

[79] Wikoff W.R., Anfora A.T., Liu J., Schultz P.G., Lesley S.A., Peters E.C., Siuzdak G. (2009). Metabolomics analysis reveals large effects of gut microflora on mammalian blood metabolites. Proc. Natl. Acad. Sci. USA.

[80] Clarke G., Grenham S., Scully P., Fitzgerald P., Moloney R.D., Shanahan F., Dinan T.G., Cryan J.F. (2012). The microbiome-gut-brain axis during early life regulates the hippocampal serotonergic system in a sex-dependent manner. Mol. Psychiatry.

[81] Clarke G., Fitzgerald P., Cryan J.F., Cassidy E.M., Quigley E.M., Dinan T.G. (2009). Tryptophan degradation in irritable bowel syndrome: Evidence of indoleamine 2,3-dioxygenase activation in a male cohort. BMC Gastroenterol..

[82] Clarke G., McKernan D.P., Gaszner G., Quigley E.M., Cryan J.F., Dinan T.G. (2012). A distinct profile of tryptophan metabolism along the kynurenine pathway downstream of toll-like receptor activation in irritable bowel syndrome. Front. Pharmacol..

[83] Fitzgerald P., Cassidy Eugene M., Clarke G., Scully P., Barry S., Quigley Eamonn M.M., Shanahan F., Cryan J., Dinan Timothy G. (2008). Tryptophan catabolism in females with irritable bowel syndrome: Relationship to interferon-gamma, severity of symptoms and psychiatric co-morbidity. Neurogastroenterol. Motil..

[84] Dehhaghi M., Kazemi Shariat Panahi H., Guillemin G.J. (2019). Microorganisms, Tryptophan Metabolism, and Kynurenine Pathway: A Complex Interconnected Loop Influencing Human Health Status. Int. J. Tryptophan Res..

[85] Waclawiková B., El Aidy S. (2018). Role of microbiota and tryptophan metabolites in the remote effect of intestinal inflammation on brain and depression. Pharmaceuticals.

[86] Gershon M.D. (2013). 5-Hydroxytryptamine (serotonin) in the gastrointestinal tract. Curr. Opin. Endocrinol. Diabetes Obes..

[87] Hata T., Asano Y., Yoshihara K., Kimura-Todani T., Miyata N., Zhang X.-T., Takakura S., Aiba Y., Koga Y., Sudo N. (2017). Regulation of gut luminal serotonin by commensal microbiota in mice. PLoS ONE.

[88] Lugo-Huitrón R., Ugalde Muñiz P., Pineda B., Pedraza-Chaverrí J., Ríos C., Pérez-De La Cruz V. (2013). Quinolinic acid: An endogenous neurotoxin with multiple targets. Oxid. Med. Cell. Longev..

[89] Terbeck S., Savulescu J., Chesterman L.P., Cowen P.J. (2016). Noradrenaline effects on social behaviour, intergroup relations, and moral decisions. Neurosci. Biobehav. Rev..

[90] Foley S., Garsed K., Singh G., Duroudier N.P., Swan C., Hall I.P., Zaitoun A., Bennett A., Marsden C., Holmes G. (2011). Impaired uptake of serotonin by platelets from patients with irritable bowel syndrome correlates with duodenal immune activation. Gastroenterology.

[91] Levy M., Kolodziejczyk A.A., Thaiss C.A., Elinav E. (2017). Dysbiosis and the immune system. Nat. Rev. Immunol..

[92] Thaiss C.A., Zmora N., Levy M., Elinav E. (2016). The microbiome and innate immunity. Nature.

[93] Wen L., Ley R.E., Volchkov P.Y., Stranges P.B., Avanesyan L., Stonebraker A.C., Hu C., Wong F.S., Szot G.L., Bluestone J.A. (2008). Innate immunity and intestinal microbiota in the development of Type 1 diabetes. Nature.

[94] Bouskra D., Brézillon C., Bérard M., Werts C., Varona R., Boneca I.G., Eberl G. (2008). Lymphoid tissue genesis induced by commensals through NOD1 regulates intestinal homeostasis. Nature.

[95] Palm N.W., De Zoete M.R., Cullen T.W., Barry N.A., Stefanowski J., Hao L., Degnan P.H., Hu J., Peter I., Zhang W. (2014). Immunoglobulin A Coating Identifies Colitogenic Bacteria in Inflammatory Bowel Disease. Cell.

[96] Kawamoto S., Tran T.H., Maruya M., Suzuki K., Doi Y., Tsutsui Y., Kato L.M., Fagarasan S. (2012). The inhibitory receptor PD-1 regulates IgA selection and bacterial composition in the gut. Science.

[97] Chang C.-H., Hsiao Y.-H., Chen Y.-W., Yu Y.-J., Gean P.-W. (2015). Social isolation-induced increase in NMDA receptors in the hippocampus exacerbates emotional dysregulation in mice. Hippocampus.

[98] Zelante T., Iannitti R.G., Cunha C., DeLuca A., Giovannini G., Pieraccini G., Zecchi R., D’Angelo C., Massi-Benedetti C., Fallarino F. (2013). Tryptophan catabolites from microbiota engage aryl hydrocarbon receptor and balance mucosal reactivity via interleukin-22. Immunity.

[99] Smith P.M., Howitt M.R., Panikov N., Michaud M., Gallini C.A., Bohlooly-Y M., Glickman J.N., Garrett W.S. (2013). The microbial metabolites, short-chain fatty acids, regulate colonic T reg cell homeostasis. Science.

[100] De Agüero M.G., Ganal-Vonarburg S.C., Fuhrer T., Rupp S., Uchimura Y., Li H., Steinert A., Heikenwalder M., Hapfelmeier S., Sauer U. (2016). The maternal microbiota drives early postnatal innate immune development. Science.

[101] Ren S., Hui Y., Obelitz-Ryom K., Brandt A.B., Kot W., Nielsen D.S., Thymann T., Sangild P.T., Nguyen D.N. (2018). Neonatal gut and immune maturation is determined more by postnatal age than by postconceptional age in moderately preterm pigs. Am. J. Physiol.-Gastrointest. Liver Physiol..

[102] Nguyen D.N., Jiang P., Frøkiær H., Heegaard P.M.H., Thymann T., Sangild P.T. (2016). Delayed development of systemic immunity in preterm pigs as a model for preterm infants. Sci. Rep..

[103] Durandy A. (2003). Ontogeny of the Immune System. Transfus. Med. Hemotherapy.

[104] Bercik P., Denou E., Collins J., Jackson W., Lu J., Jury J., Deng Y., Blennerhassett P., MacRi J., McCoy K.D. (2011). The intestinal microbiota affect central levels of brain-derived neurotropic factor and behavior in mice. Gastroenterology.

[105] Cawthon C.R., de La Serre C.B. (2018). Gut bacteria interaction with vagal afferents. Brain Res..

[106] Gareau M.G., Jury J., MacQueen G. (2008). Probiotic treatment of rat pups normalises corticosterone release and ameliorates colonic dysfunction induced by maternal separation (Gut (2007) 56, (1522–1528)). Gut.

[107] Farzi A., Fröhlich E.E., Holzer P. (2018). Gut Microbiota and the Neuroendocrine System. Neurotherapeutics.

[108] Warner B.B. (2019). The contribution of the gut microbiome to neurodevelopment and neuropsychiatric disorders. Pediatr. Res..

[109] Tamana S.K., Tun H.M., Konya T., Chari R.S., Field C.J., Guttman D.S., Becker A.B., Moraes T.J., Turvey S.E., Subbarao P. (2021). Bacteroides-dominant gut microbiome of late infancy is associated with enhanced neurodevelopment. Gut Microbes.

[110] Agrawal S., Rao S.C., Bulsara M.K., Patole S.K. (2018). Prevalence of autism spectrum disorder in preterm infants: A meta-Analysis. Pediatrics.

[111] Xie S., Heuvelman H., Magnusson C., Rai D., Lyall K., Newschaffer C.J., Dalman C., Lee B.K., Abel K. (2017). Prevalence of Autism Spectrum Disorders with and without Intellectual Disability by Gestational Age at Birth in the Stockholm Youth Cohort: A Register Linkage Study. Paediatr. Perinat. Epidemiol..

[112] Maroney D.I. (2003). Commentary Recognizing the Potential Effect of Stress and Trauma on Premature Infants in the NICU: How are Outcomes Affected?. J. Perinatol..

[113] Seki D., Mayer M., Hausmann B., Pjevac P., Giordano V., Goeral K., Unterasinger L., Klebermaß-Schrehof K., De Paepe K., Van de Wiele T. (2021). Aberrant gut-microbiota-immune-brain axis development in premature neonates with brain damage. Cell Host Microbe.

[114] Hsiao E.Y., McBride S.W., Hsien S., Sharon G., Hyde E.R., McCue T., Codelli J.A., Chow J., Reisman S.E., Petrosino J.F. (2013). Microbiota modulate behavioral and physiological abnormalities associated with neurodevelopmental disorders (La microbiota modula la fisiología intestinal y las anomalías conductuales asociadas con el autismo). Cell.

[115] Ming X., Chen N., Ray C., Brewer G., Kornitzer J., Steer R.A. (2018). A Gut Feeling: A Hypothesis of the Role of the Microbiome in Attention-Deficit/Hyperactivity Disorders. Child Neurol. Open.

[116] Pärtty A., Kalliomäki M., Wacklin P., Salminen S., Isolauri E. (2015). A possible link between early probiotic intervention and the risk of neuropsychiatric disorders later in childhood: A randomized trial. Pediatr. Res..

[117] Prehn-Kristensen A., Zimmermann A., Tittmann L., Lieb W., Schreiber S., Baving L., Fischer A. (2018). Reduced microbiome alpha diversity in young patients with ADHD. PLoS ONE.

[118] Aarts E., Ederveen T.H.A., Naaijen J., Zwiers M.P., Boekhorst J., Timmerman H.M., Smeekens S.P., Netea M.G., Buitelaar J.K., Franke B. (2017). Gut microbiome in ADHD and its relation to neural reward anticipation. PLoS ONE.

[119] Cao X., Lin P., Jiang P., Li C. (2013). Characteristics of the gastrointestinal microbiome in children with autism spectrum disorder: A systematic review. Shanghai Arch. Psychiatry.

[120] Adams J.B., Johansen L.J., Powell L.D., Quig D., Rubin R.A. (2011). Gastrointestinal flora and gastrointestinal status in children with autism-comparisons to typical children and correlation with autism severity. BMC Gastroenterol..

[121] Wang L., Christophersen C.T., Sorich M.J., Gerber J.P., Angley M.T., Conlon M.A. (2012). Elevated fecal short chain fatty acid and ammonia concentrations in children with autism spectrum disorder. Dig. Dis. Sci..

[122] Thomas R.H., Meeking M.M., Mepham J.R., Tichenoff L., Possmayer F., Liu S., MacFabe D.F. (2012). The enteric bacterial metabolite propionic acid alters brain and plasma phospholipid molecular species: Further development of a rodent model of autism spectrum disorders. J. Neuroinflamm..

[123] Nilsson N.E., Kotarsky K., Owman C., Olde B. (2003). Identification of a free fatty acid receptor, FFA2R, expressed on leukocytes and activated by short-chain fatty acids. Biochem. Biophys. Res. Commun..

[124] Nosarti C., Reichenberg A., Murray R.M., Cnattingius S., Lambe M.P., Yin L., MacCabe J., Rifkin L., Hultman C.M. (2012). Preterm Birth and Psychiatric Disorders in Young Adult Life. Arch. Gen. Psychiatry.

[125] Schwarz E., Maukonen J., Hyytiäinen T., Kieseppä T., Orešič M., Sabunciyan S., Mantere O., Saarela M., Yolken R., Suvisaari J. (2018). Analysis of microbiota in first episode psychosis identifies preliminary associations with symptom severity and treatment response. Schizophr. Res..

[126] Castro-Nallar E., Bendall M.L., Pérez-Losada M., Sabuncyan S., Severance E.G., Dickerson F.B., Schroeder J.R., Yolken R.H., Crandall K.A. (2015). Composition, taxonomy and functional diversity of the oropharynx microbiome in individuals with schizophrenia and controls. PeerJ.

[127] Hintz S.R., Kendrick D.E., Vohr B.R., Poole W.K., Higgins R.D. (2005). Changes in Neurodevelopmental Outcomes at 18 to 22 Months’ Corrected Age Among Infants of Less Than 25 Weeks’ Gestational Age Born in 1993–1999. Pediatrics.

[128] Salhab W.A., Perlman J.M., Silver L., Broyles R.S. (2004). Necrotizing Enterocolitis and Neurodevelopmental Outcome in Extremely Low Birth Weight Infants <1000 g. J. Perinatol..

[129] Bedrick A.D. (2004). Necrotizing Enterocolitis: Neurodevelopmental “Risky Business”. J. Perinatol..

[130] Siggers R.H., Siggers J., Thymann T., Boye M., Sangild P.T. (2011). Nutritional modulation of the gut microbiota and immune system in preterm neonates susceptible to necrotizing enterocolitis. J. Nutr. Biochem..

[131] Neu J. (2020). Necrotizing Enterocolitis: A Multi-omic Approach and the Role of the Microbiome. Dig. Dis. Sci..

[132] Morowitz M.J., Poroyko V., Caplan M., Alverdy J., Liu D.C. (2010). Redefining the Role of Intestinal Microbes in the Pathogenesis of Necrotizing Enterocolitis. Pediatrics.

[133] Claud E.C., Walker W.A. (2001). Hypothesis: Inappropriate colonization of the premature intestine can cause neonatal necrotizing enterocolitis. FASEB J..

[134] Jiang P., Sangild P.T., Siggers R.H., Sit W.H., Lee C.L., Wan J.M.F. (2011). Bacterial Colonization Affects the Intestinal Proteome of Preterm Pigs Susceptible to Necrotizing Enterocolitis. Neonatology.

[135] Cilieborg M.S., Boye M., MØlbak L., Thymann T., Sangild P.T. (2011). Preterm Birth and Necrotizing Enterocolitis Alter Gut Colonization in Pigs. Pediatr. Res..

[136] Pammi M., Cope J., Tarr P.I., Warner B.B., Morrow A.L., Mai V., Gregory K.E., Simon Kroll J., McMurtry V., Ferris M.J. (2017). Intestinal dysbiosis in preterm infants preceding necrotizing enterocolitis: A systematic review and meta-analysis. Microbiome.

[137] Neu J., Pammi M. (2018). Necrotizing enterocolitis: The intestinal microbiome, metabolome and inflammatory mediators. Semin. Fetal Neonatal Med..

[138] Zozaya C., García González I., Avila-Alvarez A., Oikonomopoulou N., Sánchez Tamayo T., Salguero E., Saenz de Pipaón M., García-Muñoz Rodrigo F., Couce M.L. (2020). Incidence, Treatment, and Outcome Trends of Necrotizing Enterocolitis in Preterm Infants: A Multicenter Cohort Study. Front. Pediatr..

[139] Mathipa M.G., Thantsha M.S. (2017). Probiotic engineering: Towards development of robust probiotic strains with enhanced functional properties and for targeted control of enteric pathogens. Gut Pathog..

[140] Sodhi C.P., Neal M.D., Siggers R., Sho S., Ma C., Branca M.F., Prindle T., Russo A.M., Afrazi A., Good M. (2012). Intestinal Epithelial Toll-Like Receptor 4 Regulates Goblet Cell Development and Is Required for Necrotizing Enterocolitis in Mice. Gastroenterology.

[141] Hackam D.J., Sodhi C.P., Good M. (2019). New insights into necrotizing enterocolitis: From laboratory observation to personalized prevention and treatment. J. Pediatr. Surg..

[142] Morgan R.L., Preidis G.A., Kashyap P.C., Weizman A.V., Sadeghirad B., Chang Y., Florez I.D., Foroutan F., Shahid S., Zeraatkar D. (2020). Probiotics Reduce Mortality and Morbidity in Preterm, Low-Birth-Weight Infants: A Systematic Review and Network Meta-analysis of Randomized Trials. Gastroenterology.

[143] Patel R.M., Myers L.S., Kurundkar A.R., Maheshwari A., Nusrat A., Lin P.W. (2012). Probiotic Bacteria Induce Maturation of Intestinal Claudin 3 Expression and Barrier Function. Am. J. Pathol..

[144] Martinez F.A.C., Balciunas E.M., Converti A., Cotter P.D., De Souza Oliveira R.P. (2013). Bacteriocin production by Bifidobacterium spp. A review. Biotechnol. Adv..

[145] Seghesio E., De Geyter C., Vandenplas Y. (2021). Probiotics in the Prevention and Treatment of Necrotizing Enterocolitis. Pediatr. Gastroenterol. Hepatol. Nutr..

[146] Sharif S., Meader N., Oddie S.J., Rojas-Reyes M.X., McGuire W. (2020). Probiotics to prevent necrotising enterocolitis in very preterm or very low birth weight infants. Cochrane Database Syst. Rev..

[147] Sanidad K.Z., Zeng M.Y. (2020). LOS in The Dysbiotic Gut. Cell Host Microbe.

[148] Mai V., Torrazza R.M., Ukhanova M., Wang X., Sun Y., Li N., Shuster J., Sharma R., Hudak M.L., Neu J. (2013). Distortions in Development of Intestinal Microbiota Associated with Late Onset Sepsis in Preterm Infants. PLoS ONE.

[149] Shaw A.G., Sim K., Randell P., Cox M.J., McClure Z.E., Li M.S., Donaldson H., Langford P.R., Cookson W.O.C.M., Moffatt M.F. (2015). Late-Onset Bloodstream Infection and Perturbed Maturation of the Gastrointestinal Microbiota in Premature Infants. PLoS ONE.

[150] Taft D.H., Ambalavanan N., Schibler K.R., Yu Z., Newburg D.S., Deshmukh H., Ward D.V., Morrow A.L. (2015). Center Variation in Intestinal Microbiota Prior to Late-Onset Sepsis in Preterm Infants. PLoS ONE.

[151] Stoll B.J., Hansen N.I., Adams-Chapman I., Fanaroff A.A., Hintz S.R., Vohr B., Higgins R.D. (2004). Neurodevelopmental and growth impairment among extremely low-birth-weight infants with neonatal infection. JAMA.

[152] Glass H.C., Bonifacio S.L., Chau V., Glidden D., Poskitt K., Barkovich A.J., Ferriero D.M., Miller S.P. (2008). Recurrent postnatal infections are associated with progressive white matter injury in premature infants. Pediatrics.

[153] Strunk T., Inder T., Wang X., Burgner D., Mallard C., Levy O. (2014). Infection-induced inflammation and cerebral injury in preterm infants. Lancet. Infect. Dis..

[154] Sewell E., Roberts J., Mukhopadhyay S. (2021). Association of Infection in Neonates and Long-Term Neurodevelopmental Outcome. Clin. Perinatol..

[155] Venkatesh K.K., Leviton A., Hecht J.L., Joseph R.M., Douglass L.M., Frazier J.A., Daniels J.L., Fry R.C., O’Shea T.M., Kuban K.C.K. (2020). Histologic chorioamnionitis and risk of neurodevelopmental impairment at age 10 years among extremely preterm infants born before 28 weeks of gestation. Am. J. Obstet. Gynecol..

[156] Mitha A., Foix-L’Hélias L., Arnaud C., Marret S., Vieux R., Aujard Y., Thiriez G., Larroque B., Cambonie G., Burguet A. (2013). Neonatal Infection and 5-year Neurodevelopmental Outcome of Very Preterm Infants. Pediatrics.

[157] Mukhopadhyay S., Puopolo K.M., Hansen N.I., Lorch S.A., DeMauro S.B., Greenberg R.G., Cotten C.M., Sánchez P.J., Bell E.F., Eichenwald E.C. (2020). Impact of Early-Onset Sepsis and Antibiotic Use on Death or Survival with Neurodevelopmental Impairment at 2 Years of Age among Extremely Preterm Infants. J. Pediatr..

[158] Simonsen K.A., Anderson-Berry A.L., Delair S.F., Dele Davies H. (2014). Early-onset neonatal sepsis. Clin. Microbiol. Rev..

[159] Rodriguez-Granger J., Alvargonzalez J.C., Berardi A., Berner R., Kunze M., Hufnagel M., Melin P., Decheva A., Orefici G., Poyart C. (2012). Prevention of group B streptococcal neonatal disease revisited. The DEVANI European project. Eur. J. Clin. Microbiol. Infect. Dis..

[160] Puopolo K.M., Draper D., Wi S., Newman T.B., Zupancic J., Lieberman E., Smith M., Escobar G.J. (2011). Estimating the Probability of Neonatal Early-Onset Infection on the Basis of Maternal Risk Factors. Pediatrics.

